# Association of pre-season musculoskeletal screening and functional testing with sports injuries in elite female basketball players

**DOI:** 10.1038/s41598-019-45773-0

**Published:** 2019-06-26

**Authors:** Laimonas Šiupšinskas, Toma Garbenytė-Apolinskienė, Saulė Salatkaitė, Rimtautas Gudas, Vytenis Trumpickas

**Affiliations:** 1 Department of Sports Medicine, Medical Academy, Lithuanian University of Health Sciences, Tilžės street 18, Kaunas, 47181 Lithuania; 2 Sports Trauma and Arthroscopic Unit, Hospital of Lithuanian University of Health Sciences, Eivenių street 2, Kaunas, 50161 Lithuania; 3 Gijos clinic AB ‘Ortopedijos technika’, Partizanų g, 17/Savanorių pr.284, Kaunas, 49476 Lithuania

**Keywords:** Preventive medicine, Population screening

## Abstract

Basketball is one of the most popular sports in Lithuania, and participation in women’s basketball is on the rise. Pre-participation examinations, including musculoskeletal screening and functional performance testing, is an essential part of a multidisciplinary approach to prevent future injuries. Because the lower extremities are the most commonly-injured body area in basketball players. Assessing fundamental movement qualities is of utmost importance. The aim of our study was to determine if functional tests can predict sports injuries in elite female basketball players. A total of 351 records for professional female basketball players were screened during 2013–2016 season. We analysed functional characteristics before the season and used functional performance tests for injury risk assessment: the Functional Movement Screen (FMS), the lower quarter Y Balance test (YBT-LQ) and the Landing Error Scoring System (LESS). Data from 169 players’ records were analysed: 77 of them made it to the end of season without injury, making up the non-injured group, while 92 of them suffered lower limb sport injuries during the sport season (injury group). Student’s t-test and the Mann-Whitney U-test were used to determine differences between groups. The most commonly encountered sports injuries in our population were those of knee 40.2% and ankle 38%. The injury group had a lower total FMS score (*p* = 0.0001) and higher total LESS score (*p* = 0.028) than non-injury group. The dynamic balance of lower limbs was similar in both groups. Imperfect functional movement patterns and poor jump-landing biomechanics during pre-season screening were associated with lower extremity injuries in elite female basketball players. Impairments of dynamic stability in the lower extremities were not associated with injury rates in our population. A combination of functional tests can be used for injury risk evaluation in female basketball players.

## Introduction

The popularity of basketball is on the rise, with an estimated 11% of the world’s population (450 million people) currently playing basketball in 213 countries affiliated with the International Basketball Federation (FIBA)^1^. Not only is the popularity of basketball increasing, but also the intensity with which it is played. The physiological demands of the sport include elevated aerobic and anaerobic capacities in addition to the integration of physical characteristics such as muscle strength, power, endurance, flexibility, speed, agility, and skill. Frequent jumping, landing and changes of direction make up much of the physical load of competitive games, with players exposed to high levels of eccentric loading^2^.

Playing any sport comes with a considerable probability of injury for elite athletes^3,4^ and basketball players in particular, both amateur and professional, are at high risk. In terms of the various body part groups (e.g. head and waist, upper extremities or lower extremities), much of the literature addressing basketball injuries mentions the lower extremities as the most likely to be injured^5–8^.

The highest incidence is seen in adolescents, and the incidence is 3–5 times higher in female than male athletes^9,10^. Studies of professional female basketball players in the United States have shown that they sustain 60% more injuries^11^. The dangers and risks associated with playing basketball may result not only in serious injury, but may also seriously impair athletes’ ability to earn a living or to engage in other professional, social and recreational activities, and impact their overall quality of life in general.

A key component of athletic preparation is pre-season musculoskeletal screening and testing^12–15^. The National Athletic Trainers’ Association (NATA)^12^ highlights musculoskeletal injury as a common cause of reduced sports activities (i.e., a loss of training and game time).

Some authors claim that Functional Movement Screen tests (FMS), the Y Balance test for the lower quarter (YBT-LQ) and the Landing Error Scoring System for the jump-landing task (LESS) are popular in-the-field sport medicine screening tools, all able to identify players at risk of injury^16,17^. Each of these assessments makes it easy to identify inefficient and/or compensatory movement tendencies, useful at the end of rehabilitation to determine an athlete’s readiness to play sports again. Screening is of interest to injury researchers, physical therapists/coaches, strength and conditioning specialists and sports medicine practitioners^18–20^.

The aim of our study was to determine the degree of association of such functional tests with sports injuries in elite female basketball players. We hypothesized that poor functional tests results during pre-season screenings would indicate an increased risk of sports injuries in female basketball players.

## Materials and Methods

### Participants

**Figure 1:**
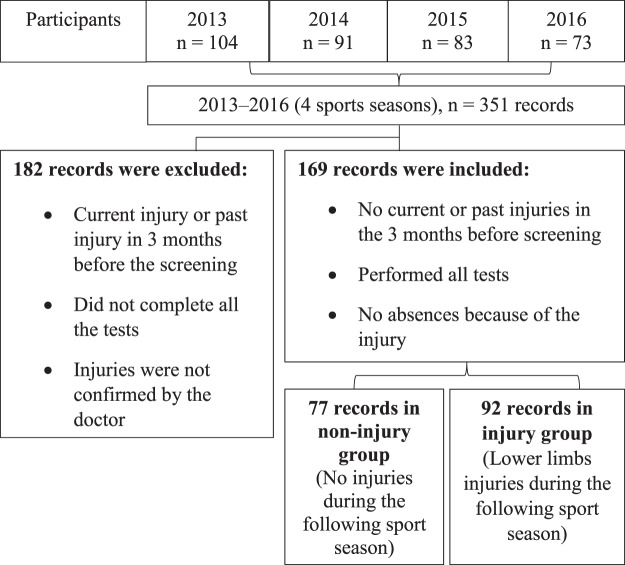
Study design.

The X women’s basketball league (XWBL) represented the first division of the X women’s basketball championship and included the top 8 X women’s basketball teams, each with 12–14 players. The basketball season generally lasts about 7 months. All teams were asked to participate in the survey. 351 XWBL players were screened over a period of 4 years (September 2013 to September - 2016). The study design shown in Fig. 1.

### Pre-season screening

Pre-season screening looked at the entire body; however, our study was concerned with the lower limb only, as most injuries occurred in the lower extremities^21^. Assessing an athlete’s movement capacity or ability to perform fundamental movements related to athletic performance was considered by many authors to be a more appropriate examination of an athlete’s potential injury risk and readiness to train/compete^10,15,22^. All measurements were taken from 2013 to 2016, one month before the beginning of the regular *X* Championship season, at each team’s training base. Pre-season evaluation consisted of the Y balance test (YBT-LQ), functional movement screen (FMS) and the landing error scoring system (LESS). Two experienced sports physiotherapists, each with more than five years of clinical practice and research expertise, scored all participants during all trials.

The FMS is a comprehensive tool which was used to assess the quality of fundamental movement patterns, thereby identifying an individual athlete’s physical limitations or asymmetries. The FMS test battery, which was described by Cook *et al*.^13,14^, was specifically designed to bridge the gap between pre-season physical examinations and physical performance testing^23^. Using a 4-point scale, the FMS evaluates performance in 7 fundamental movement patterns: deep squat, hurdle step, in-line lunge, shoulder mobility, active straight leg raise, trunk stability push-up, and rotary stability. A score of 0 was issued if the subject experienced any pain during the assessment process; a score of 1 for poor performance; and 3 for excellent performance^24^. For each item, the highest score of the three trials was recorded and used to generate an overall total FMS score with a maximum value of 21^25^.

Used as a screening tool to evaluate risk of injury as described by Plisky *et al*.^26^, YBT-LQ was helpful in assessing players’ dynamic balance. This test evaluates performance during single-leg balance with reaching tasks in the anterior, posteromedial, and posterolateral directions^27^ to determine the lower extremity’s movement asymmetry and balance deficits^26,28^. Participants were asked to stand, barefoot or wearing athletic footwear, with support foot positioned in the centre of a platform, positioned just against a starting line. While maintaining this unipodal stance, the individual was then asked to reach her free limb in the three directions in relation to the static foot^26^. An overall YBT-LQ performance score was calculated by averaging the maximum reach distance in each direction (normalized according to leg length), generating the composite reach score. Absolute differences between left and right lower extremity reach distances were also examined to assess symmetry^29^.

LESS was used to evaluate whole body and lower limb biomechanics during vertical jump drop. Two digital video cameras (FujiFilm FinePix S9200) were placed in front of and to the right of the athlete to capture frontal and sagittal images of all jump-landing trials^30^. Each participant started the trial by standing on a 30-cm-high box placed half their body height away from a landing area, indicated by a line marked on the ground. Athletes were instructed to jump forward from the box (with both limbs moving in unison), land on the ground just over the line, and then jump as high as possible immediately after landing^31,32^. The LESS score is simply a count of landing technique ‘errors’ on a range of readily observable human movements: knee flexion at initial contact, hip flexion at initial contact, trunk flexion at initial contact, ankle plantar flexion at initial contact, medial knee position at initial contact, lateral trunk flexion at initial contact, stance width (wide, narrow), foot position (external and internal rotation), symmetric initial foot contact at initial contact, knee-flexion displacement, hip-flexion displacement, trunk-flexion displacement, medial-knee displacement, joint displacement and overall impression, for a total of 12 scored items^32^. The higher the LESS score, the poorer the landing technique after a jump. The LESS scores were then analysed as a continuous variable as well as according to the categorical scale defined by its developers as poor (>6), moderate (≤6 to >5), good (≤5 to >4), and excellent (≤ 4). The LESS test was performed as described by Padua *et al*.^30^.

The teams’ sport physicians and sport physiotherapists were requested to report, on a daily basis, all injuries (acute and chronic) which occurred in competitions and/or training as well as all illnesses (or the lack of injuries/illnesses) using a specially designed, web-based injury and illnesses register called ‘*X*’. Registry was based on the International Olympic Committee questionnaire titled the ‘Daily report on injuries and illnesses’ and translated into *X*^33,34^. All injuries analysed in the study were diagnosed and confirmed by the sports medicine doctor and orthopeadic surgeon at the X Universitety of Health Science Hospital. A lower extremity injury was defined as any injury that caused the participant to miss 1 or more practices or games, diagnosed by a sport medicine doctor. An injury was registered if it occurred during a scheduled basketball game or practice session and stopped a player from participating in basketball training or play during the following 24 hours.

All procedures were approved by the Kaunas Regional Research Ethics Committee (no. BE-2–27) and all methods were performed in accordance with the relevant guidelines and regulations. Players signed a written informed consent form before participation. Informed consent was obtained from all individual participants included in the study.

### Statistical analysis

Statistical analysis was done with SPSS 22 Software (IBM Corp., Armonk, NY, USA). Differences for normally-distributed independent samples were assessed using Student’s t test results presented as a mean (95% confidence intervals (CI)). Differences for independent samples which were not normally distributed were assessed using the Mann-Whitney U test and presented as a median (minimum-maximum). All statistical procedures *p* < 0.05 were regarded as significant.

## Results

Data from 169 players were analysed: 92 of them sustained lower limb sports injuries during the same season. As such, we analysed functional characteristics before injury and named the group ‘injury group’ (mean age = 23.1 ± 5.7 yrs.; mean weight = 71.5 ± 9.3 kg; mean height = 180.2 ± 7.7 cm). The data were compared with 77 players who were injury-free during the following athletic season, called the ‘non-injury group’ (mean age = 23.2 ± 5.7 yrs.; mean weight = 70.1 ± 8.5 kg; mean height = 179.5 ± 7.3 cm). No significant differences in anthropometric characteristics were found between the groups.

The most common musculoskeletal injuries in our study were those of the anterior cruciate ligament (ACL), the medial collateral ligament (MCL), and the lateral collateral ligament (LCL) of the knee (21.7%, n = 20); acute ankle ligament injuries (15.2%, n = 14); chronic ankle ligament tendinopathy (14.1%, n = 13), knee cartilage injuries, and meniscal injuries (13%, n = 12). 40.2% of all injuries were in the knee while 38% were in the ankle (Table 1). The parameters of lower extremity dynamic balance is shown in Table 2. The study failed to find a statistically significant difference between injured and non-injured players. Athletes from the injured group scored 1.3 point lower on their total FMS score and 1 point higher on their total LESS score than non-injured players (14.1 vs 15.4 for FMS, respectively, *p* = 0.0001; and 8 vs 7 for LESS, respectively, *p* = 0.017) (Tables 3 and 4).

**Table 1:** Lower limb injuries in professional female basketball players 2013–2016.

Injuries	n	%
Knee ACL, MCL, LCL injuries	20	21.7
Acute ankle ligaments injuries	14	15.2
Chronic ankle ligaments tendinopathy	13	14.1
Knee cartilage, meniscus injuries	12	13
Chronic patellar tendinopathy	6	6.5
Acute ankle fractures	5	5.4
Thigh muscle injuries	4	4.4
Ankle stress fractures	3	3.3
Knee dislocation, instability	3	3.3
Achilles tendon tendinopathy	3	3.3
Groin pain	2	2.2
Foot stress fractures	2	2.2
Foot fasciitis	2	2.2
Knee fractures	1	1.1
Hip fractures	1	1.1
Knee arthritis, bursitis	1	1.1

**Table 2:** Results of lower quarter Y balance test.

	Non-injured group	Injured group	P value
Composite score of left leg	102.9 (95% CI: 101.2; 104.6)	103.6 (95% CI: 102.2; 105)	0.543
Composite score of right leg	103.4 (95% CI: 101.4; 105.4)	103.1 (95% CI: 101.7; 104.5)	0.809
Difference between left and right composite scores	−0.5 (95% CI: -1.7; 0.6)	0.5 (95% CI: −0.1; 1.1)	0.138
Anterior difference	0.9 (95% CI: −0.1; 1.8)	0.39 (95% CI: −0.5; 1.3)	0.463
Posteromedial difference	−1.1 (95% CI: −3.5; 1.4)	0.8 (95% CI: −0.2; 1.7)	0.172
Posterolateral difference	−0.8 (95% CI: −1.9; 0.2)	0.0 (95% CI: −0.9; 1,0)	0.235

**Table 3:** Total FMS scores.

	Non-injured group	Injured group	P value
FMS score (mean)	15.4 (95% CI: 15; 15.9)	14.1 (95% CI: 13.6; 14.7)	0.0001

**Table 4:** Total LESS scores.

	Non-injured group	Injured group	P value
LESS score (median)	7 (1; 16)	8 (1; 13)	0.017

## Discussion

Women’s participation in basketball has increased in recent years, bringing with it increased awareness of health and medical issues specific to female athletes. As such, we analyzed the physical conditions of female basketball players before injury, with the aim of then determining the degree of association between the cited functional tests and sports injuries in elite female basketball players.

Injuries to lower extremities are common in team sports such as basketball (58–66%)^35,36^. In our study, we found that knee and ankle injuries (40.2% and 38%, respectively) were the most-commonly diagnosed injuries in female basketball players, accounting for approximately 78.2% of all lower limb injuries. Prodromes *et al*.^37^ highlighted that nearly half (45%) of all ankle injuries occurred during landing, with another third (30%) sustained during cutting (twist/turn) manoeuvres. For instance, up to 16% of female basketball players will injury their ACL during their professional athletic career^37^. Findings identify basketball injuries as a significant public health problem and emphasize that greater efforts need to be directed toward their prevention.

Dynamic balance is an integral factor in lower extremity injuries^26^. Screening for dynamic balance competence has been proposed as a potential way to prevent injuries^26–28,38^. One recent systematic review of measurement properties and injury correlation by Hegedus *et al*.^39^ reported that there is strong evidence that modified three-direction SEBT/YBT tests can accurately identify injury risk in field and court athletes, with both a composite reach score difference of less than 94% and an anterior reach difference of 4 cm or greater being associated with increased injury risk. In our study, pre-season YBT-LQ evaluation showed that composite scores in both groups were higher than the injury risk level cut-off point. Also, anterior reach difference scores were less than 4 cm.

Our results showed that lower extremity dynamic stability impairment was not associated with injury rates in our population. Myklebust *et al*.^40^ also failed to find differences in dynamic balance between injured legs and those of healthy players. We could posit that, in our study, composite scores were higher because players were at an advanced competition level.

Research on the use and suitability of the FMS is the subject of much debate. While multiple studies have found the FMS to be an invalid screening tool^41–43^, others have demonstrated a significant relationship between FMS scores and injury occurrence^23,44^. In our study, there was a significant difference between FMS scores in injured and non-injured groups. As shown in Table 2, the injured group in this study was very close to the pre-established injury cut-off point of 14^23,44–46^.

Kiesel *et al*.^44^ evaluated the ability of the FMS to predict injury. They found that players with an FMS score of 14 or less had an 11-times greater risk of injury and a 51% increased probability of incurring a serious injury during the season than those scoring 15 or higher. In a study of female collegiate soccer, volleyball and basketball players, Chorba *et al*.^23^, found that athletes scoring 14 or less had a 69% higher injury rate and a 4-fold increased risk of injury. Additionally, a score of 15 or below was correlated to an injury rate 56% higher than those with scores of 16 or above, and a score of 13 or less resulted in an injury rate as high as of 81%. It could thus be stated that poor functional movement patterns are associated with lower limb injury.

Most studies involving LESS were conducted to identify high-risk movement patterns, leaving athletes more vulnerable to lower extremity injury^47^. One of the goals of our study was to determine if the total LESS score was greater in individuals who had a lower limb injury, compared with healthy, non-injured players. Both groups demonstrated ‘poor’ landing techniques (i.e. scored >6 points, where a higher LESS score indicates poor technique in jump landing)^30^ and both groups may be at increased risk of injury. Screening how an athlete lands is an important consideration. Most recently, Padua *et al*. reported that elite youth soccer players with LESS scores of five or more had a higher risk of ACL injuries than athletes with LESS scores below five, suggesting that five was the optimal cut-off point for elite youth field and court athletes^32^.

Movement screenings such as the YBT, FMS and LESS have been proposed as methods to identify at-risk individuals^13,26,48,49^. This clinical commentary offers evidence-informed choices for a battery of musculoskeletal screens and functional performance tests used by the authors which are specific to basketball. Future research in this area should assess a more detailed evaluation of fatigue, overuse, chronic injuries and sport-specific risk factors.

## Limitations

Several limitations for this study should be noted. First, our results are not injury specific: they can be generalized for individuals with symptomatic concomitant injuries, but not for ACL injuries or asymptomatic concomitant injuries. Second, data from four seasons were analyzed in general. Furthermore, data was not collected after the sports season ended.

## Conclusion

Faulty functional movement patterns and poor jump landing biomechanics during pre-season screening were associated with lower extremity injuries in elite female basketball players. Lower extremity dynamic stability impairment was not associated with higher injury rates in our population. A combination of functional tests can be used for injury risk evaluation in female basketball players.
